# Cholesterol Reprogramming in Acute Myeloid Leukemia: Integrating Tumor-Intrinsic Metabolism and Immune Crosstalk

**DOI:** 10.3390/diseases14070246

**Published:** 2026-07-07

**Authors:** Francisco Alejandro Lagunas-Rangel

**Affiliations:** Department of Genetics and Molecular Biology, Centro de Investigación y de Estudios Avanzados del Instituto Politécnico Nacional, Av. Instituto Politécnico Nacional 2508, San Pedro Zacatenco, Gustavo A. Madero, Mexico City 07360, Mexico; alejandr030.lagunas@outlook.com

**Keywords:** LDLR, HMGCR, immune checkpoint proteins, antitumor activity, statins

## Abstract

Acute myeloid leukemia (AML) is a genetically and biologically heterogeneous hematologic neoplasm that arises from the clonal transformation of hematopoietic progenitor cells. AML cells undergo extensive metabolic reprogramming to sustain proliferation, survival, and adaptation to therapeutic stress. Among these alterations, cholesterol metabolism has emerged as a critical determinant of leukemic cell fitness. AML cells enhance cholesterol biosynthesis, uptake, trafficking, and storage, generating a dynamic network that supports membrane organization, mitochondrial function, oncogenic signaling, and resistance to therapy. Beyond these tumor-intrinsic roles, accumulating evidence indicates that cholesterol and its metabolites actively shape communication between leukemic and immune cells, influencing immune checkpoint expression, inflammatory signaling, and antitumor immune responses within the bone marrow microenvironment. This narrative review examines the mechanisms underlying cholesterol reprogramming in AML and discusses how alterations in cholesterol homeostasis integrate metabolic adaptation with immune regulation. Particular emphasis is placed on the interplay between cholesterol metabolism, leukemic stem cell persistence, therapeutic resistance, and immune dysfunction. Emerging therapeutic strategies targeting cholesterol-related pathways are also considered. Collectively, these findings position cholesterol metabolism as a central interface between tumor-intrinsic biology and immune crosstalk, highlighting its potential as a therapeutic vulnerability in AML.

## 1. Introduction

Acute myeloid leukemia (AML) is a group of hematological disorders caused by the clonal transformation of hematopoietic precursors through the acquisition of chromosomal rearrangements and multiple genetic mutations that confer advantages in terms of proliferation and survival, as well as having a negative effect on differentiation [1]. AML accounts for approximately 28% of leukemia cases worldwide and is the most common acute leukemia in adults. Despite advances in the molecular understanding of AML and the development of targeted therapies, clinical outcomes remain poor for many patients, and the disease continues to be associated with substantial mortality [2]. Consequently, there is a growing interest in identifying metabolic vulnerabilities that contribute to leukemogenesis and may serve as therapeutic targets.

A common feature of many types of cancer is the reprogramming of cholesterol metabolism, defined as the coordinated alteration of the pathways involved in the biosynthesis, uptake, storage, transport and excretion of cholesterol, which enables cancer cells to meet the metabolic and signaling requirements of malignant growth [3]. In this sense, cancer cells require elevated cholesterol levels to maintain membrane biogenesis and other essential cellular functions [4]. For example, cholesterol is a major component of lipid rafts, specialized microdomains of the plasma membrane that organize signaling complexes. In cancer, lipid rafts regulate processes such as cell growth, adhesion, migration, invasion, and apoptosis, thereby shaping cellular responses to external signals [5]. Furthermore, cholesterol biosynthetic intermediates, such as farnesyl pyrophosphate (FPP) and geranylgeranyl pyrophosphate (GGPP), are essential for the prenylation of oncogenic proteins, including RAS, which promote the survival and proliferation of tumor cells [6]. Similarly, cholesterol-derived metabolites, such as the oncometabolite 6-oxo-cholestan-3b,5a-diol, promote tumor growth by activating glucocorticoid receptors [7]. Consistent with these observations, both preclinical and clinical studies demonstrate that targeting cholesterol metabolism can inhibit tumor growth, remodel the immune microenvironment, and restore effective antitumor immune responses [8].

Beyond its tumor-intrinsic functions, cholesterol metabolic reprogramming also suppresses antitumor immunity and contributes to the establishment of a microenvironment that favors tumor progression [9,10]. Cholesterol accumulation and altered lipid metabolism influence multiple immune cell populations, including T lymphocytes, macrophages, dendritic cells, and natural killer cells [10]. These alterations can promote immune exhaustion, suppressive phenotypes, and dysfunctional immune signaling, highlighting the central role of cholesterol metabolism in the crosstalk between malignant cells and the immune system [9,10].

Importantly, AML presents a unique biological context compared with solid malignancies. Unlike solid tumors, AML arises from hematopoietic cells that inherently participate in immune regulation and circulate systemically rather than forming a localized tumor mass [10]. Consequently, alterations in cholesterol metabolism may influence not only leukemic cell fitness but also immune communication within the bone marrow niche and systemic antileukemic responses.

Despite growing evidence linking cholesterol metabolism to AML pathogenesis, current knowledge remains fragmented, particularly regarding how cholesterol-dependent pathways coordinate leukemic cell metabolism and immune regulation. A more comprehensive understanding of these interconnected processes may reveal novel therapeutic opportunities and improve our understanding of AML biology. In this way, this narrative review examines the role of cholesterol metabolism in AML, highlighting how cholesterol reprogramming supports leukemic cell survival, metabolic adaptation, and therapeutic resistance. Emerging evidence indicating that cholesterol and its metabolites influence immune communication within the leukemic microenvironment, thereby shaping immune evasion and antitumor activity, is also discussed. By integrating tumor-intrinsic metabolic mechanisms with immune crosstalk, this review highlights cholesterol metabolism as a central regulator of AML pathogenesis and discusses its potential as a therapeutic vulnerability.

## 2. Cholesterol Homeostasis: Synthesis, Uptake, and Systemic Transport

Cholesterol is an organic compound belonging to the steroid family, defined by a rigid structure composed of four fused hydrocarbon rings, a hydrophilic hydroxyl group at position 3, and a hydrophobic aliphatic tail extending from position 17 [11]. This compound has several biological functions, including regulating membrane fluidity and permeability, acting as a precursor to steroid hormones, corticosteroids, bile acids and vitamins, and modulating genetic transcription [12].

Cholesterol is derived from two main sources: endogenous synthesis and dietary intake. De novo biosynthesis is a tightly regulated, multi-step metabolic pathway that occurs primarily in the liver, starting from acetyl-CoA. Acetyl-CoA is converted to 3-hydroxy-3-methylglutaryl-CoA (HMG-CoA) by HMG-CoA synthase (HMGCS1 in the cytosol, HMGCS2 in the mitochondria), then reduced to mevalonate by HMG-CoA reductase (HMGCR). Mevalonate is subsequently phosphorylated and decarboxylated to form isopentenyl pyrophosphate (IPP), a key isoprenoid precursor. IPP is converted to farnesyl pyrophosphate (FPP) by farnesyl pyrophosphate synthase (FDPS), and two molecules of FPP are condensed by squalene synthase (SQS) to produce squalene. Squalene is then oxidized by squalene monooxygenase (SQLE) to 2,3-epoxysqualene and cyclized by lanosterol synthase (LSS) to form lanosterol, which is finally converted to cholesterol via the Bloch and/or Kandutsch-Russell pathways [3,13].

In contrast, dietary cholesterol is absorbed by intestinal enterocytes via the Niemann-Pick type 1 C1 transporter (NPC1L1), with adaptor proteins such as NUMB and LIM domain-binding protein 1 (LIMA1) facilitating internalization and transport. It is then transported to the liver as chylomicrons or as free cholesterol. In hepatocytes, cholesterol is oxidized by cytochrome P450 (CYP) enzymes or reactive oxygen species (ROS) to form oxysterols or esterified by sterol O-acyltransferases (SOATs). Cholesterol and its esters are packaged into very low-density lipoproteins (VLDL), which are hydrolyzed by lipoprotein lipase (LPL) into intermediate-density lipoproteins (IDL) and subsequently into low-density lipoproteins (LDL). Approximately 75% of plasma cholesterol is transported by LDL and the remainder by high-density lipoproteins (HDL). Around 70% of LDL is cleared by the liver via LDL receptor (LDLR)-mediated endocytosis, and the rest is taken up by peripheral tissues [14].

## 3. Cholesterol in AML Patients

**Figure 1:**
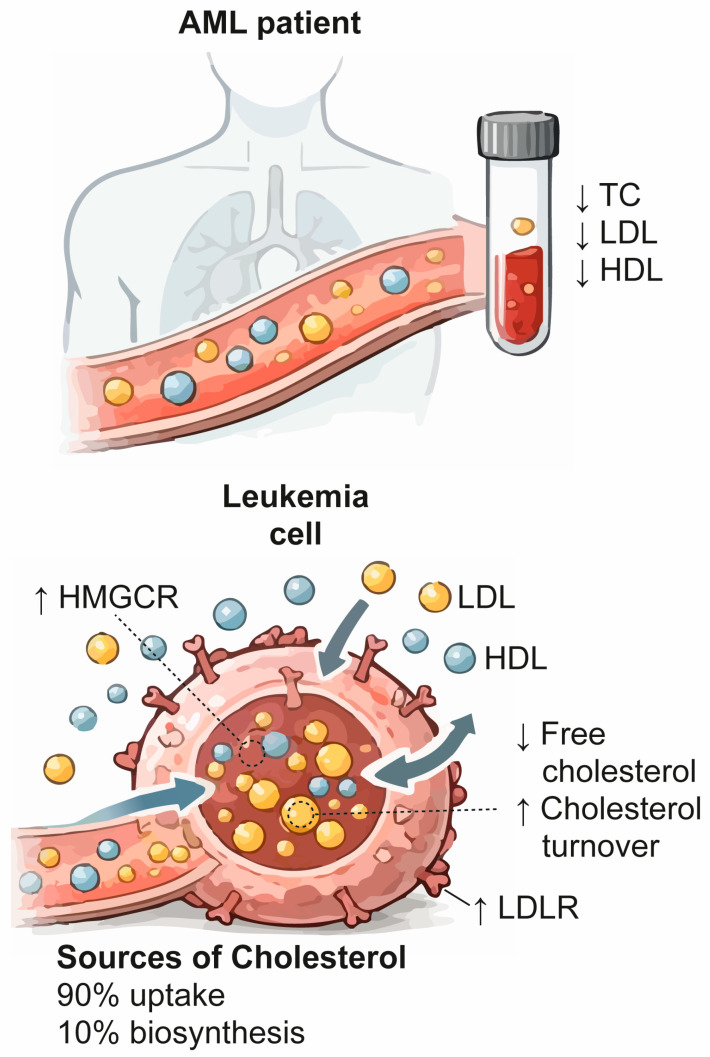
**Disruption of cholesterol homeostasis in AML.** At diagnosis, patients with AML typically have reduced circulating levels of total cholesterol, LDL, and HDL compared to healthy individuals, while triglyceride and VLDL levels remain virtually unchanged. After achieving complete remission, cholesterol levels generally increase to those observed in healthy controls, suggesting a partial normalization of systemic lipid metabolism with disease control. At the cellular level, AML blasts tend to have a strong dependence on cholesterol. This is due to a significant increase in receptor-mediated LDL uptake, which is the main source of intracellular cholesterol, along with an increase in endogenous cholesterol synthesis. Despite this, intracellular free cholesterol remains lower than in normal hematopoietic cells, which could indicate an accelerated cholesterol turnover to support rapid proliferation and metabolic demands.

Some plasma lipidomic studies in AML samples have reported reduced plasma cholesterol levels, with no significant differences between sexes (Figure 1) [15,16,17]. This decrease appears to be partly due to lower levels of cholesterol esters. Furthermore, plasma concentrations of 7α-hydroxy-4-cholesten-3-one, a biomarker of bile acid synthesis, were notably lower in AML patients compared to healthy controls. In this regard, reduced bile acid production may affect intestinal cholesterol absorption, thus contributing to the hypocholesterolemia observed in AML patients [17]. Notably, lower plasma cholesterol levels have been reported to be associated with worse overall survival (2.67 mmol/L as the cutoff value) [15].

In addition to total cholesterol levels, the relative proportions of circulating lipoproteins also influence the progression of AML. HDL particles have been associated with more favorable clinical outcomes in AML, whereas elevated LDL levels may support leukemic progression by increasing cholesterol availability to malignant cells [18]. In this context, HDLs may function as metabolic inhibitors of leukemic progression by promoting cholesterol efflux via reverse cholesterol transport, thus limiting the intracellular availability of cholesterol. Their anti-inflammatory and antioxidant properties may support immune cell function and preserve antileukemic responses [19]. Conversely, LDLs can facilitate AML progression by transporting cholesterol to leukemic cells via LDLR-mediated uptake. Oxidized LDL can further enhance inflammatory signaling and metabolic stress, contributing to a leukemia-friendly environment [20].

At the time of diagnosis, patients with AML have approximate total cholesterol, LDL, and HDL levels of 137 mg/dL (3.5 mmol/L), 87 mg/dL (2.3 mmol/L), and 20 mg/dL (0.5 mmol/L), respectively [21]. Triglyceride and VLDL levels remain comparable to those of healthy controls [22].

It is worth noting that these lipid parameters increase after achieving complete remission, approaching the levels observed in healthy individuals (total cholesterol generally between 150 and 200 mg/dL [3.9 to 5.2 mmol/L], LDL cholesterol between 70 and 130 mg/dL [1.8 to 3.4 mmol/L], and HDL cholesterol between 40 and 60 mg/dL [1.0 to 1.6 mmol/L]), suggesting a partial normalization of the lipid profile associated with disease control [21].

The degree of hypocholesterolemia also varies across AML subtypes. According to the French–American–British (FAB) classification (Table 1), the lowest plasma cholesterol levels were observed in poorly differentiated AMLs, including M1, as well as in leukemias with a monocytic component, particularly M5a. While these subtypes were also associated with higher white blood cell counts compared to other FAB categories, low cholesterol levels were independently related to both FAB subtype and white blood cell count. Notably, remission was accompanied by a significant increase in cholesterol levels in patients who, at the time of diagnosis, had low cholesterol or elevated white blood cell counts [23,24]. Furthermore, the induction of myeloid differentiation in AML cells is closely related to a marked suppression of cholesterol biosynthesis [25].

In AML cells, cellular cholesterol acquisition is drastically accelerated across both entry pathways. Receptor-mediated LDL uptake and degradation—the source of approximately 90% of cellular cholesterol—is markedly upregulated, exhibiting a 3- to 100-fold increase in AML mononuclear cells compared to healthy controls [26,27]. Concurrently, de novo cholesterol synthesis from acetate, which provides the remaining 10%, displays a 2- to 30-fold elevation [26]. This increased clearance of circulating lipids by leukemic blasts is thought to contribute significantly to the systemic hypocholesterolemia observed in AML patients [22].

Despite this enhanced external supply, AML cell lines and primary blasts exhibit a striking depletion of intracellular free cholesterol, with levels approximately 50% lower than those observed in normal hematopoietic mononuclear cells [28,29]. This marked reduction suggests a state of highly accelerated cholesterol turnover. Instead of accumulating in its non-esterified form, free cholesterol appears to be rapidly used or redistributed to meet the demanding needs of rapid cell growth, energy metabolism, and proliferative activity [26]. This metabolic acceleration is not uniform across all leukemias, being especially pronounced in subtypes with a monocytic component, such as FAB-M4 and FAB-M5 [27].

Furthermore, primary AML cells exhibit distinct intracellular trafficking, partitioning significantly greater amounts of cholesterol into cell membranes, lipid droplets, and mitochondria compared to normal hematopoietic cells—a structural redistribution closely correlated with heightened disease aggressiveness [30]. This altered cholesterol handling persists and adapts under therapeutic pressure. For instance, chemotherapy-resistant AML blasts demonstrate a markedly higher accumulation of intracellular cholesterol than their treatment-sensitive counterparts [31]. To maintain this high lipid flux while simultaneously preventing cholesterol-induced lipotoxicity, AML cells strictly regulate the intracellular fate of the molecule. Any residual free cholesterol not immediately consumed through metabolic turnover is rapidly converted into cholesteryl esters [32]. Consequently, while systemic plasma levels of these esters are depleted due to continuous cellular uptake, over 90% of the intracellular cholesterol pool within leukemic cells is maintained in its esterified form [29], effectively neutralizing lipotoxic stress while securing a safe metabolic reservoir that promotes cell survival.

Collectively, these findings suggest a metabolic paradox in AML (Figure 1), characterized by systemic hypocholesterolemia despite increased cellular cholesterol demand and turnover within leukemic blasts.

**Table 1:** **Cholesterol metabolic alterations across FAB subtypes of AML.**

FAB Subtype	Systemic Cholesterol Status	Alterations in Cholesterol Metabolism	Representative Genetic Alterations	Hypothetical Links of Gene Alterations with Cholesterol Metabolism	References
M0 Minimally differentiated AML	Profound systemic hypocholesterolemia	Constitutive mevalonate pathway activation (HMGCR/HMGCS1 upregulation), promoting self-renewal and leukemic immaturity	Biallelic CEBPA mutations frequently co-occurring with TET2 or WT1 mutations	CEBPA-p30-driven activation of de novo cholesterol biosynthesis	[33,34,35]
M1 AML without maturation	Severe plasma hypocholesterolemia	Enhanced cholesterol biosynthesis and lipid raft stabilization	Biallelic CEBPA mutations with recurrent NPM1 mutations and FLT3-ITD insertions.	CEBPA-p30-driven activation of de novo cholesterol biosynthesis	[33,35,36]
M2 AML with maturation	Moderate systemic plasma hypocholesterolemia	Coordinated lipid metabolic reprogramming, altering ceramide–sphingolipid synthesis and membrane lipid raft organization	RUNX1::RUNX1T1 fusion [t(8;21)(q22;q22.1)]	RUNX1-RUNX1T1 remodels the epigenome and blocks differentiation. Cholesterol-rich membrane rafts stabilize CXCR4 and FLT3 signaling, supporting leukemic cell survival	[23,33]
M3 Acute promyelocytic leukemia (APL)	Frequent systemic dyslipidemia Hypertriglyceridemia Normal or elevated total cholesterol	Lipophagy activation and impaired lipid homeostasis, promoting fatty acid supply for mitochondrial β-oxidation	PML::RARA fusion [t(15;17)(q24;q21)]	PML-RARA sequesters RXR from PPARγ, repressing lipid transport genes and promoting resistin and PCSK9 secretion; cooperation with PPARα at super-enhancers induces FLT3 transcription under high-fat conditions	[37,38]
M4 Acute myelomonocytic leukemia	Severe systemic hypocholesterolemia	Enhanced LDLR-mediated LDL uptake, altered sphingolipid–ceramide metabolism, and oxidized LDL-driven M4-like macrophage polarization	CBFB::MYH11 fusion [inv(16)(p13.1q22)] with recurrent NPM1 mutations and FLT3-ITD insertions.	CBFB-MYH11 rewires membrane sphingolipid biosynthesis, whereas NPM1 mutations promote Commander/Retriever-dependent recycling of endosomal receptors, including LDLR	[23,39,40]
M4Eo Acute myelomonocytic leukemia with eosinophilia					
M5 Acute monocytic leukemia	The most extreme systemic plasma hypocholesterolemia of all subtypes.	Cytokine-driven LDLR overexpression (TNF-α, IL-6 and IL-8), promoting sterol-independent LDL uptake and cholesterol acquisition	KMT2A::MLLT3 fusion [t(9;11)(p21.3;q23.3)], occasionally co-occurring with FLT3-ITD mutations	KMT2A-MLLT3 drives ACSL4 dependency for polyunsaturated lipid synthesis and storage, while FLT3-ITD activates AKT to stabilize SREBP1/2, enhancing FASN expression and lipogenesis	[23,41,42,43]
M5a Acute monoblastic leukemia					
M5b Acute monocytic leukemia (differentiated)					
M6 Acute erythroid leukemia	Marked plasma hypocholesterolemia	Impaired repression of ABCA1 and LDLR during differentiation, resulting in excessive cholesteryl ester accumulation	Loss-of-function mutations or deletions in GATA1 or its essential cofactor ZFPM1 (FOG1)	GATA1–FOG1 represses SREBP2 during normal myeloid differentiation, limiting cholesterol-driven mTORC1 signaling and cell-cycle progression; loss of this axis in AML-M6 impairs enucleation	[23,44,45]
M7 Acute megakaryoblastic leukemia	Moderate systemic plasma hypocholesterolemia	m6A-dependent regulation of lipid-metabolism transcripts, promoting aberrant lipid accumulation and incomplete differentiation	RBM15::MKL1 fusion [t(1;22)(p13.3;q13.3)]	RBM15 reshapes m6A methylation of metabolic transcripts, whereas MKL1 loss promotes aberrant megakaryoblastic adipogenic programming and maturation arrest through PRMT1-dependent coactivation	[23,46]

## 4. Reprogramming of Cholesterol Metabolism in AML Cells

In healthy cells, cholesterol levels are tightly regulated at both the cellular and systemic levels by coordinated transcriptional programs.

The main regulators of cholesterol homeostasis are sterol regulatory element-binding protein 2 (SREBP2), liver X receptors (LXRs), and nuclear factor erythroid 2-related factor 1 (NRF1) [47]. When intracellular cholesterol or cholesterol-derived oxysterols accumulate, the SREBP2 pathway is suppressed through sterol-dependent retention of the SREBP2–sterol regulatory element-binding protein cleavage-activating protein (SCAP) complex in the endoplasmic reticulum. This retention is mediated by the interaction of SCAP with insulin-induced gene proteins (INSIGs), resulting in reduced cholesterol biosynthesis and uptake [48]. Simultaneously, desmosterol, the immediate precursor of cholesterol in the Bloch biosynthetic pathway, along with oxysterols, activates LXRs. LXR activation induces the expression of genes involved in cholesterol efflux and clearance, including ATP-binding cassette subfamily A member 1 (ABCA1), ATP-binding cassette subfamily G member 1 (ABCG1), ATP-binding cassette subfamily G member 5 (ABCG5), and ATP-binding cassette subfamily G member 8 (ABCG8), as well as additional regulators such as myosin regulatory light chain interacting protein (MYLIP) [49]. Elevated cholesterol levels also inhibit the nuclear translocation of NRF1, thereby relieving its repression of the LXR pathway and further promoting cholesterol efflux. Conversely, under conditions of cholesterol depletion, these regulatory pathways act in a coordinated but opposing manner to enhance cholesterol biosynthesis and absorption while limiting cholesterol efflux and esterification, ultimately restoring cholesterol homeostasis [3].

**Figure 2:**
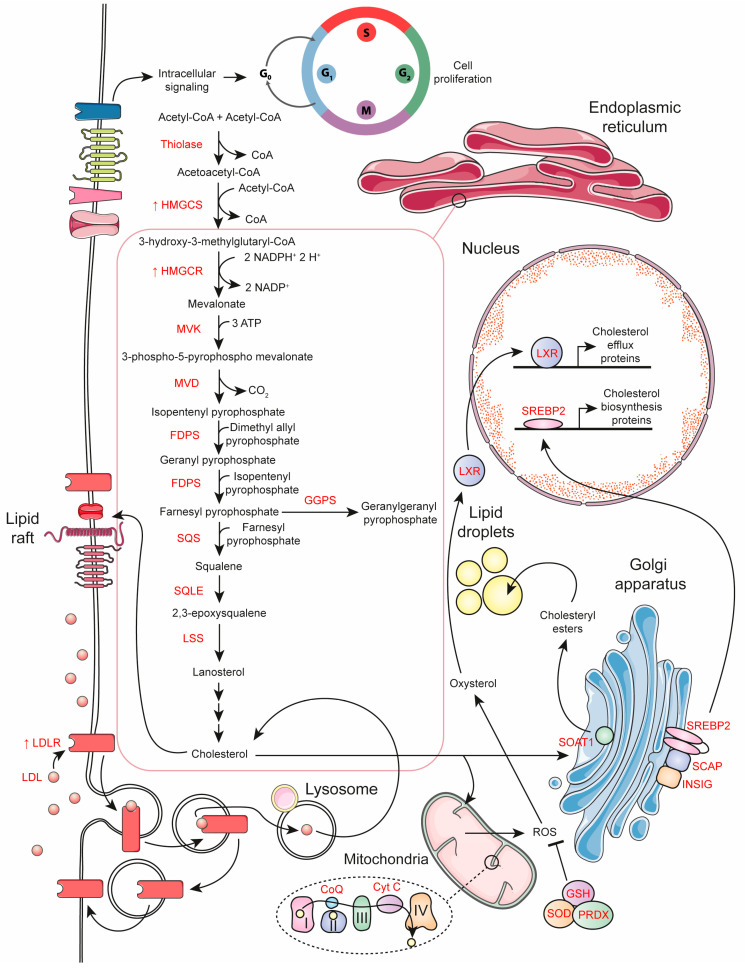
**Reprogramming of cholesterol metabolism in AML.** In AML, leukemic cells undergo extensive reprogramming of cholesterol metabolism, with increased de novo synthesis and uptake from the circulation. Acquired cholesterol is rapidly allocated to membrane biogenesis, including lipid raft formation, organelle growth, and vesicular trafficking, as well as mitochondrial membrane remodeling to protect against apoptosis. Excess cholesterol is esterified and stored in lipid droplets, while oxysterol production is tightly regulated. This metabolic adaptation promotes proliferation, survival, and chemoresistance. CoA: coenzyme A; HMGCS: 3-hydroxy-3-methylglutaryl-CoA synthase; HMGCR: 3-hydroxy-3-methylglutaryl-CoA reductase; NADPH: reduced nicotinamide adenine dinucleotide phosphate; NADP^+^: oxidized nicotinamide adenine dinucleotide phosphate; ATP: adenosine triphosphate; CO_2_: carbon dioxide; FDPS: farnesyl diphosphate synthase; MVK: mevalonate kinase; MVD: mevalonate diphosphate decarboxylase; SQS: squalene synthase; SQLE: squalene epoxidase; LSS: lanosterol synthase; GGPS: geranylgeranyl diphosphate synthase; SOAT1: sterol O-acyltransferase 1; SREBP2: sterol regulatory element-binding protein 2; SCAP: SREBP cleavage-activating protein; INSIG: insulin-induced gene protein; LDLR: low-density lipoprotein receptor; LDL: low-density lipoprotein; Cyt c: cytochrome c; CoQ: coenzyme Q (ubiquinone); ROS: reactive oxygen species; GSH: reduced glutathione; SOD: superoxide dismutase; PRDX: peroxiredoxins; LXR: liver X receptor.

AML is characterized by a coordinated reprogramming of cholesterol metabolism compared to healthy hematopoietic cells (Figure 2). The transcriptional profile reveals upregulation of pathways involved in cholesterol uptake and de novo biosynthesis, while genes mediating cholesterol efflux or negative feedback regulation remain virtually unaltered or repressed. This imbalance indicates impaired cholesterol homeostasis control in AML cells [33].

At the plasma membrane level, AML cells exploit functional LDLRs to achieve highly efficient uptake and metabolism of both native and modified LDL particles [50]. Unlike healthy cells, however, AML blasts exhibit severely impaired sterol-mediated feedback control, leading to persistently aberrant LDLR expression that sustains a high-volume cholesterol influx to drive proliferation [51]. This receptor-mediated internalization is further amplified by autocrine and paracrine signaling loops driven by leukemic cell-derived factors, such as tumor necrosis factor α (TNF-α) [52]. Consistent with this increased LDLR activity, plasma cholesterol levels at diagnosis show an inverse correlation with LDLR expression in malignant cells. These findings further support the idea that the hypocholesterolemia observed in AML patients is primarily due to excessive LDL uptake by leukemic blasts [53]. Paralleling this influx machinery, scavenger receptor class B type 1 (SR-B1), a key mediator of cellular cholesterol uptake and redox homeostasis, is concurrently overexpressed in AML cells, where its elevated status serves as an independent marker associated with adverse clinical outcomes [54].

Regarding cholesterol biosynthesis, AML exhibits pronounced molecular and metabolic heterogeneity. Typically, HMGCS1, the first enzyme of the de novo cholesterol synthesis pathway, is overexpressed in both newly diagnosed and relapsed or refractory AML. This upregulation is frequently driven by the direct binding of GATA1 to the HMGCS1 promoter, which enhances transcription and subsequently promotes leukemic cell survival by preserving mitochondrial and endoplasmic reticulum integrity under cellular stress [55]. However, a distinct metabolic profile emerges in the substantial subset of AML patients harboring mutations in the methylcytosine dioxygenase TET2. In these specific cases, TET2 deficiency and its subsequent loss of enzymatic activity lead to the downregulation of HMGCS1, as wild-type TET2 normally maintains HMGCS1 expression by catalyzing DNA demethylation within its promoter region. Consequently, the mevalonate pathway is suppressed, creating a genotype-specific metabolic vulnerability that makes TET2-deficient tumor cells highly sensitive to statin-induced apoptosis [56].

Another key enzyme in cholesterol biosynthesis is HMGCR, which is generally overexpressed in AML, but appears to play different roles depending on the differentiation state of the leukemic cells. In HL-60 models, forcing granulocytic differentiation with dimethyl sulfoxide (DMSO) induces a profound suppression of cholesterol biosynthesis, marked by reduced HMGCR activity and diminished incorporation of acetate and mevalonate into sterol intermediates. Conversely, prompting monocyte/macrophage differentiation via phorbol 12-myristate 13-acetate (PMA)—despite halting cell proliferation—triggers a dose-dependent upregulation of HMGCR activity, accelerating biosynthetic flux and elevating intracellular cholesterol pools. This divergence aligns with the physiological traits of mature leukocytes, where peripheral blood monocytes preserve active synthesis while mature granulocytes lose post-squalene sterol-producing capacity [42]. Crucially, this lineage-specific metabolic wiring offers a plausible mechanistic framework that may help explain the clinical observations noted earlier, suggesting a potential basis for the exceptionally high intracellular cholesterol demand and accelerated turnover characteristic of monocytic and myelomonocytic AML subtypes (FAB-M4 and FAB-M5) [27].

Higher cholesterol levels upregulate FMS-like tyrosine kinase 1 (FLT-1) expression, enhance placenta growth factor (PlGF)/vascular endothelial growth factor (VEGF)–induced leukemia cell viability, and stimulate VEGF production by AML cells. Within the bone marrow, cholesterol enables FLT-1 to concentrate in cholesterol-rich membrane domains, where it associates with caveolin-1, thereby promoting activation of survival and proliferative VEGF signaling pathways [29].

Altogether, AML cells bypass normal homeostatic controls to drive a dual upregulation of cholesterol synthesis and uptake (Figure 2). This metabolic reprogramming is dynamically modulated by genetic and lineage heterogeneity to sustain leukemic survival and proliferation, offering distinct, context-specific vulnerabilities for therapeutic targeting [57].

### Oncogenic Control of Cholesterol Metabolism in AML Cells

Cholesterol metabolism in cancer cells is tightly controlled by the balance between oncogenic signaling and tumor suppressor pathways. Activation of oncogenes enhances cholesterol biosynthesis, uptake, and esterification to meet the demands of rapid proliferation and survival. Conversely, tumor suppressors restrict these metabolic programs to preserve cellular homeostasis. During tumorigenesis, the loss of tumor suppressor function disrupts this regulatory balance, resulting in uncontrolled cholesterol metabolic activity that promotes malignant growth [3].

AML is characterized by extensive genetic and epigenetic heterogeneity, including numerous mutations, chromosomal alterations (such as deletions, gains, translocations, and inversions), and epigenetic modifications [58]. Notably, several of these alterations reprogram cholesterol metabolism through distinct mechanisms across AML subtypes.

For example, AML cases with the t(4;11) translocation, which results in a fusion between the histone-lysine N-methyltransferase 2A (KMT2A) and AF4/FMR2 family member 1 (AFF1) genes, exhibit significantly higher expression of SREBF2 compared to AML patients with a normal karyotype and healthy bone marrow samples [59]. Similarly, internal tandem duplication of FMS-like tyrosine kinase 3 (FLT3-ITD) reprograms lipid metabolism by activating SREBPs, which are upregulated in FLT3-ITD-positive AML compared to FLT3 wild-type AML [60]. FLT3-ITD is a common activating mutation in AML and is associated with a poor prognosis [1,61]. In an independent cohort of patients with cytogenetically normal AML (CN-AML), SREBF2 overexpression was significantly associated with reduced overall survival and event-free survival. Comprehensive genomic and transcriptomic analyses revealed that SREBF2 interacts with multiple molecular regulatory elements that contribute to the pathogenesis of CN-AML. These SREBF2-associated alterations included co-expression of leukemia-related genes, dysregulation of immune pathways, changes in microRNA expression and DNA methylation patterns, as well as structural variants affecting the first exon and the 5′ untranslated region (5′UTR) [62]. Collectively, these findings identify SREBF2 as a recurrent metabolic regulator across genetically distinct AML subtypes.

Beyond SREBF2, additional AML-associated transcription factors have been implicated in the regulation of cholesterol biosynthesis and mevalonate pathway activity. In AML, high expression of the homeobox transcription factor A5 (HOXA5) predicts poor overall survival and is frequently associated with mutations in FLT3 and nucleophosmin 1 (NPM1). Mechanistically, HOXA5 promotes leukemic cell survival and proliferation by positively regulating the transcription of genes in the mevalonate-cholesterol biosynthetic pathway, thereby increasing intracellular cholesterol levels necessary for membrane organization and oncogenic signaling [63]. Likewise, a recent preprint identified a cholesterol-dependent metabolic phenotype in AML with biallelic CCAAT/enhancer-binding protein α (CEBPA) mutations and concurrent TET2 or WT1 loss-of-function alterations. Predominance of the oncogenic CEBPA-p30 isoform promoted cholesterol biosynthesis and mevalonate pathway dependency, sensitizing leukemic cells to cholesterol synthesis inhibitors [35]. CEBPA findings illustrate how disruption of transcription factors with tumor suppressor functions can contribute to cholesterol metabolic rewiring in AML. Taken together, these findings raise the possibility that various transcription factors associated with AML may also converge in the regulation of cholesterol biosynthesis.

Compared with isocitrate dehydrogenase 1 (IDH1) wild-type AML cells, AML cells carrying the R132H mutation show increased expression of proteins involved in cholesterol and sterol biosynthesis, including isopentenyl diphosphate delta isomerase 1 (IDI1), LSS, and emopamil-binding protein (EBP). Despite this upregulation, IDH1 R132H mutant cells displayed an altered cholesterol distribution, characterized by reduced levels of esterified cholesterol and a compensatory increase in the proportion of free cholesterol relative to wild-type cells [47]. These findings suggest that mutant IDH1 not only affects cholesterol biosynthesis but also alters intracellular cholesterol trafficking and storage.

In acute promyelocytic leukemia (APL), the PML-retinoic acid receptor α (RARα) fusion oncoprotein is generated by the chromosomal translocation t(15;17). The pseudokinase Tribbles homolog 3 (TRIB3) interacts directly with PML-RARα, promoting the degradation and inhibition of the peroxisome proliferator-activated receptor γ (PPARγ). TRIB3-mediated inhibition of PPARγ disrupts lipid homeostasis, whereas cooperative interactions between PPARα and PML-RARα at super-enhancer regions support proliferative transcriptional programs [37,38]. Given the central role of PPAR family members in lipid and cholesterol homeostasis, these findings suggest that PML-RARα-associated transcriptional programs may indirectly influence cholesterol metabolic networks.

Overall, these observations suggest that diverse AML-associated genetic alterations converge on cholesterol metabolic pathways, supporting cholesterol biosynthesis and homeostasis as common vulnerabilities across molecular AML subtypes. The cholesterol-related metabolic characteristics associated with each AML subtype are summarized in Table 1.

## 5. Functional Cholesterol Pools in AML Cells

AML cells do not process cholesterol through a single metabolic pathway. Instead, they distribute cholesterol among distinct functional pools, including plasma membrane cholesterol, mitochondrial cholesterol, cholesteryl ester stores, and signaling-associated cholesterol. This compartmentalization allows leukemic cells to withstand high proliferative and metabolic demands while avoiding the toxic effects of excess free cholesterol.

Rapidly proliferating AML cells require cholesterol to maintain continuous membrane synthesis, which supports plasma membrane lipid rafts, organelle biogenesis, and vesicular trafficking. Rather than being stored, cholesterol is allocated to these functional needs, making it essential for cell proliferation and progression throughout the cell cycle, particularly the S phase [64].

Compared to normal cells, AML blasts exhibit a greater abundance of lipid rafts, which provide platforms for the clustering of signaling proteins that drive tumor growth and survival [63]. Cholesterol-rich lipid rafts organize receptors, kinases, and adaptor proteins into signaling platforms by regulating membrane order, fluidity, and protein clustering. Through this function, cholesterol controls key pathways, including receptor tyrosine kinase, Janus kinase (JAK)–signal transducer and activator of transcription (STAT), phosphoinositide 3-kinase (PI3K)–AKT serine/threonine kinase (AKT)–mechanistic target of rapamycin kinase (mTOR), G protein-coupled receptor (GPCR), Hedgehog, and Wnt/β-catenin signaling pathways [5]. Although most commonly associated with myeloproliferative neoplasms, JAK2-V617F can contribute to leukemic transformation and provides a useful example of lipid raft-dependent oncogenic signaling. Alteration of the integrity of these lipid rafts, including by statins such as simvastatin, lovastatin, and atorvastatin, inhibits JAK2-V617F signaling at micromolar concentrations, inducing growth arrest and apoptosis. JAK2-V617F-positive cells are more sensitive to statins than cells without this mutation, highlighting the kinase’s dependence on lipid rafts [65]. In addition, statins have been reported to inhibit the phosphorylation of additional kinases, thereby reducing insulin signaling, as well as the activity of the EGF-EGFR and PI3K/AKT pathways [66].

Beyond their structural and signaling roles at the plasma membrane, cholesterol pools are also redistributed to intracellular organelles, particularly mitochondria. Although AML cells exhibit aerobic glycolysis, they rely heavily on mitochondrial metabolism and oxidative phosphorylation (OXPHOS) to meet their energy and survival needs. This dependence is especially pronounced in leukemic stem cells (LSCs), which use OXPHOS as their primary energy source to maintain their survival and function [67]. In this respect, AML cells exhibit a greater mitochondrial mass without a proportional increase in respiratory chain complex activity [68]. AML cells increase cholesterol trafficking to the mitochondria, resulting in an accumulation of mitochondrial cholesterol that stabilizes the architecture of the inner mitochondrial membrane and prevents the release of cytochrome c, thus protecting the cells from apoptosis [69]. However, excessive cholesterol buildup within mitochondria can be detrimental, as it disrupts the proper assembly of respiratory chain supercomplexes, leading to increased ROS production [70]. To counteract this, AML cells exhibit strong antioxidant defenses, such as increased glutathione (GSH) synthesis and overexpression of superoxide dismutases (SOD) and peroxiredoxins (PRDX). These systems collectively protect leukemic cells from cell death induced by oxidative stress [71,72].

When cholesterol exceeds immediate structural and metabolic requirements, AML cells redirect it toward storage pools. In AML cells, excess cytoplasmic free cholesterol is esterified by sterol O-acyltransferase 1 (SOAT1) into cholesteryl esters and stored in lipid droplets [73]. Regulated hydrolysis of cholesteryl esters (for example via carboxylesterase 1 [CES1]) allows for the release of cholesterol on demand, maintaining a mobilizable pool and preventing the accumulation of free cholesterol. Therefore, cholesteryl esters act as a buffer, protecting AML cells from cholesterol-induced lipotoxicity [16]. AML cells utilize CES1-mediated hydrolysis of cholesterol esters to meet their metabolic and proliferative needs [74]. Beyond its metabolic role, high CES1 expression has also been associated with CNS relapse and poor clinical outcome in AML. Mechanistically, CES1 expression is associated with immunosuppressive M2 macrophages and poor clinical outcomes [75].

A small fraction of cholesterol is converted into oxysterols, which are involved in metabolic regulation and signaling [76]. Oxysterols act as endogenous LXR ligands, activating LXR-dependent transcriptional programs that enhances cholesterol efflux [77]. Oxysterol levels are tightly controlled, as an excess of oxysterols can be cytotoxic. In K562 cells, exposure to micromolar concentrations of free cholesterol or oxysterols induces programmed cell death, while cholesterol esters remain non-toxic. Mechanistically, oxysterols directly alter membrane integrity, triggering apoptosis [78]. Similarly, in the U-937 and HL-60 cell lines, 7β-hydroxycholesterol (7β-OHC) at 30 µM rapidly induces apoptosis within hours, whereas 25-hydroxycholesterol (25-OHC) at the same concentration mainly exerts a cytostatic effect without triggering cell death [79]. In THP-1 cells, both 7β-OHC and 25-OHC inhibit proliferation at low micromolar concentrations by inducing apoptosis and causing cell cycle arrest in the G2/M phase [80]. Clinically, AML patients with high expression of cytochrome P450 7B1 (CYP7B1), which promotes the metabolism and clearance of 27-hydroxycholesterol (27-OHC), show significantly shorter survival compared to patients with low expression of CYP7B1, who are expected to maintain higher intracellular levels of 27-OHC [81].

Taken together, these findings indicate that by distributing cholesterol into distinct functional compartments, leukemic cells simultaneously promote proliferation, mitochondrial function, resistance to apoptosis, and oncogenic signaling, while minimizing cholesterol-induced toxicity.

## 6. Cholesterol-Driven Chemoresistance Mechanisms in AML Cells

Increasing evidence indicates that chemotherapy actively drives adaptive cholesterol remodeling in AML cells. Rather than serving as a passive consequence of treatment, therapeutic stress reprograms intracellular cholesterol trafficking, redistributing cholesterol among distinct functional pools. This process is characterized by increased mitochondrial cholesterol accumulation, maintenance of cholesterol-rich plasma membrane domains that sustain pro-survival signaling, and enhanced esterification and storage of excess cholesterol as cholesteryl esters within lipid droplets [82]. Collectively, these metabolic adaptations preserve cellular homeostasis under chemotherapeutic stress, promote leukemic cell survival, and contribute to the acquisition of chemoresistance.

Accordingly, cholesterol levels increase in NB4 and HL60 cells treated with cytarabine (Ara-C), daunorubicin (DNR), or γ-irradiation. These AML cells rapidly—within 24 h—mount substantial increases in intracellular cholesterol in response to therapeutic stress [31]. Mechanistically, these drug treatments significantly increase the abundance and activity of LDLR and HMGCR in most AML cells, indicating that increased cholesterol uptake along with increased de novo synthesis contributes to cholesterol accumulation during drug exposure [83]. Likewise, Ara-C-resistant AML cells also overexpress the multifunctional lipid transporter CD36/fatty acid translocase (FAT) [84]. Within the hypoxic bone marrow microenvironment, activation of cystathionine β-synthase (CBS)-dependent hydrogen sulfide (H_2_S) signaling promotes Ara-C resistance in AML cells by driving cholesterol-rich metabolic adaptations. Specifically, CBS-H_2_S signaling induces thrombospondin-1 (THBS1) methylation and silencing, activating the fatty acid transporter CD36 [85]. CD36-mediated lipid uptake increases cholesterol and promotes its accumulation within mitochondrial membranes, where elevated cholesterol content decreases membrane fluidity and enhances mitochondrial resistance to stress-induced damage. These adaptations preserve mitochondrial integrity under chemotherapeutic stress, thereby supporting leukemic cell survival and contributing to drug resistance [86,87].

Besides increasing cholesterol uptake and biosynthesis, drug-resistant AML cells also enhance cholesterol esterification through sirtuin-3 (SIRT3)-dependent metabolic rewiring. SIRT3 contributes to treatment resistance by regulating FAO, particularly through a compensatory mechanism that increases cholesterol biosynthesis and esterification. This elevated cholesterol metabolism protects leukemic stem cells from excessive lipid accumulation and the resulting lipotoxicity. Treatment with YC8-02, a selective SIRT3 inhibitor, sensitizes AML cells to venetoclax, but not to cytarabine or doxorubicin. This specificity is due to the fact that venetoclax resistance is partly due to increased fatty acid metabolism, which SIRT3 helps to maintain [32]. In addition, elevated intracellular cholesterol has been associated with biomechanical adaptations in drug-resistant AML cells, including increased cellular stiffness, higher expression of cytoskeletal proteins, and activation of PPAR signaling, suggesting that cholesterol-dependent metabolic rewiring also influences the physical properties associated with treatment resistance [88].

Although cholesterol remodeling promotes chemoresistance, it also creates exploitable metabolic vulnerabilities. Ara-C- and doxorubicin (DOX)-resistant THP-1 cells display marked dependence on the mevalonate-cholesterol biosynthetic pathway, requiring multiple enzymes including HMGCR, mevalonate kinase (MVK), phosphomevalonate kinase (PMVK), mevalonate diphosphate decarboxylase (MVD), SQS, squalene epoxidase (SQLE), and LSS for survival [89]. Consequently, inhibition of cholesterol biosynthesis selectively compromises resistant leukemic cells, particularly within the hypoxic bone marrow niche. Consistent with this vulnerability, statins enhance the cytotoxic activity of Ara-C in multiple AML models [84]. Although 12-O-tetradecanoylphorbol 13-acetate (TPA) is primarily used as an experimental differentiation agent rather than a standard AML therapy, resistance to TPA is likewise associated with profound alterations in sterol metabolism. HL-60 cells resistant to TPA exhibit marked accumulation of the sterol intermediates lanosterol and dihydrolanosterol, whereas TPA-sensitive cells fail to accumulate sterols and instead release phospholipids following treatment [70].

Although dysregulated cholesterol metabolism is a common feature of both AML and acute lymphoblastic leukemia (ALL), its biological and therapeutic implications differ substantially [90]. In ALL, glucocorticoids are a cornerstone of therapy, and altered lipid metabolism has been implicated in glucocorticoid resistance through modulation of membrane cholesterol, glucocorticoid receptor signaling and pro-survival pathways [91]. By contrast, AML is intrinsically refractory to glucocorticoids and instead relies heavily on enhanced de novo cholesterol synthesis and LDL uptake to support leukemic growth and adaptation to chemotherapy-induced stress [3]. At present, there is no direct evidence that cholesterol metabolism drives glucocorticoid resistance in AML. However, the constitutive activation of the mevalonate pathway and its downstream signaling networks may indirectly contribute to the steroid-resistant phenotype. Whether pharmacological inhibition of cholesterol metabolism can restore glucocorticoid sensitivity in AML remains an open question that warrants further investigation.

Collectively, these studies indicate that cholesterol metabolism is not merely associated with chemoresistance but actively shapes the adaptive response of AML cells to therapeutic stress. Although these adaptations initially promote survival, they also generate metabolic dependencies that may be therapeutically exploited through pharmacological inhibition of cholesterol synthesis, uptake, or storage.

## 7. Cholesterol Metabolism Reprogramming Shapes Immune Crosstalk and Antitumor Immunity in AML

**Figure 3:**
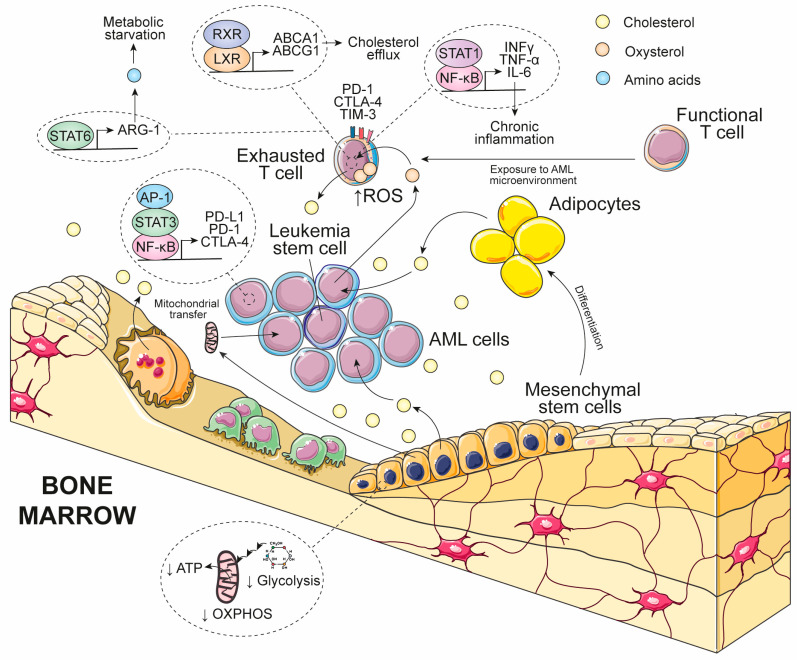
**Cholesterol metabolism reprogramming promotes immune evasion within the bone marrow microenvironment of AML.** Normal T cells derived from healthy hematopoietic stem cells (HSCs) acquire an exhausted phenotype following prolonged exposure to the AML-remodeled bone marrow microenvironment. Cholesterol and its metabolites activate transcription factors, including NF-κB, AP-1, and STAT3, promoting the expression of immune checkpoint molecules (PD-1, PD-L1, and CTLA-4) and contributing to T-cell exhaustion, accumulation of regulatory T cells, and reduced T- and natural killer (NK)-cell cytotoxicity. AML cells also establish reciprocal metabolic interactions with MSCs. Mitochondrial transfer from MSCs to AML cells supports leukemic metabolism and chemoresistance, whereas AML cells reciprocally reprogram MSC metabolism by suppressing oxidative phosphorylation and glycolysis. Cholesterol metabolites, particularly oxysterols, induce oxidative stress, activate LXR-dependent cholesterol efflux pathways, and impair mitochondrial bioenergetics within the immune microenvironment. In addition, 25-OHC inhibits mTORC1 signaling through LYCHOS, activating the AMPK–STAT6–ARG1 axis and promoting arginine depletion, thereby limiting T-cell proliferation and effector function.

The bone marrow is not only the primary site of leukemogenesis but also a metabolically active microenvironment that protects and supports AML cells. This niche comprises mesenchymal stromal cells (MSCs), adipocytes, endothelial cells, and multiple immune cell populations that are functionally reprogrammed by leukemic blasts to promote disease progression [92,93]. Beyond sustaining leukemic cell metabolism, cholesterol remodeling also reshapes bidirectional communication between AML cells and the immune microenvironment, thereby promoting immune evasion and limiting antitumor immunity (Figure 3).

One mechanism by which cholesterol metabolism contributes to immune escape is through the regulation of immune checkpoint signaling. Cholesterol and its metabolites activate transcription factors including nuclear factor κ-light-chain-enhancer of activated B cells (NF-κB), activator protein-1 (AP-1), and STAT3, which regulate the expression of immune checkpoint molecules such as programmed cell death protein 1 (PD-1), programmed death-ligand 1 (PD-L1), and cytotoxic T-lymphocyte-associated protein 4 (CTLA-4) [10]. Increased expression of these immune checkpoint molecules is associated with immune evasion and disease progression in AML [94]. Consistently, bone marrow-infiltrating CD4+ and CD8+ T cells from patients with AML display a pronounced exhausted phenotype characterized by high PD-1 expression, increased eomesodermin (EOMES), and reduced T-box transcription factor TBX21 expression. Moreover, memory T-cell populations exhibit features of terminal exhaustion, including co-expression of PD-1, CTLA-4, and T-cell immunoglobulin and mucin-domain-containing protein 3 (TIM-3), whereas relapsed patients accumulate larger numbers of regulatory T cells, reflecting a profoundly immunosuppressive microenvironment [95]. Accordingly, leukemic cells become less susceptible to T-cell- and natural killer (NK)-cell-mediated cytotoxicity [96]. Importantly, the functional relevance of cholesterol metabolism in T-cell exhaustion has recently been supported by a multicenter phase II clinical trial demonstrating that the addition of rosuvastatin to venetoclax plus azacitidine significantly reduced both PD-1+CD4+ and PD-1+CD8+ exhausted T-cell populations while enhancing T-cell cytotoxicity against leukemic cells [97]. These findings suggest that pharmacological modulation of cholesterol metabolism may not only impair leukemic cell survival but also partially restore antitumor immunity by preventing therapy-associated T-cell exhaustion.

Beyond regulating immune checkpoint signaling, AML cells also remodel the metabolic landscape of the bone marrow niche. Reciprocal metabolic interactions between AML cells and MSCs play a central role in this process. Previous studies have shown that mitochondria can be transferred from MSCs to AML blasts, supporting oxidative metabolism, leukemic proliferation, and chemotherapy resistance. Conversely, AML cells metabolically reprogram MSCs by suppressing oxidative phosphorylation and glycolysis, thereby reducing the metabolic fitness of stromal cells and reshaping the leukemic niche [98]. Although direct mitochondrial transfer from AML cells to lymphocytes has not been clearly demonstrated, metabolic dysfunction within the leukemic microenvironment is associated with increased oxidative stress and altered cholesterol metabolism. Excess ROS may promote oxysterol accumulation, whereas excessive intracellular cholesterol impairs mitochondrial oxidative phosphorylation, reducing ATP production and limiting the energetic capacity required for effective immune responses. Furthermore, oxysterols activate LXR-dependent transcriptional programs that promote cholesterol efflux, further disrupting cholesterol homeostasis and immune cell function [10].

Oxysterols also contribute to immunosuppression by altering nutrient availability within the leukemic microenvironment. In particular, 25-OHC inhibits mTORC1 signaling by competing with cholesterol for binding to the lysosomal cholesterol sensor LYCHOS. This activates AMP-activated protein kinase α (AMPKα), which enhances STAT6 signaling and upregulates arginase-1 (ARG1), ultimately promoting arginine depletion [99]. Together with the high consumption of essential amino acids by AML blasts, these mechanisms deprive infiltrating T cells of critical biosynthetic substrates required for proliferation and effector function, thereby reinforcing immune suppression within the bone marrow niche [100].

Emerging evidence further suggests that cholesterol metabolism contributes to inflammatory remodeling of the leukemic microenvironment. Cholesterol derivatives, particularly oxysterols, regulate transcriptional programs controlled by NF-κB and members of the STAT family, thereby influencing cytokine production and immune cell behavior. Consistent with this concept, AML has been associated with increased STAT1 activity, enhanced interferon-γ (IFN-γ) signaling, and elevated inflammatory risk scores that reflect persistent cytokine-driven immune activation [101]. Although the direct contribution of cholesterol metabolism to these inflammatory programs in AML remains to be fully established, these observations support the concept that cholesterol and its metabolites coordinate both inflammatory and immunosuppressive signaling pathways within the leukemic microenvironment.

Collectively, these findings indicate that cholesterol metabolism extends beyond supporting leukemic cell proliferation and survival. By regulating immune checkpoint expression, remodeling stromal metabolism, impairing immune cell bioenergetics, promoting nutrient deprivation, and shaping inflammatory signaling, cholesterol and its metabolites orchestrate multiple mechanisms of immune evasion that collectively suppress antitumor immunity and favor AML progression (Figure 3).

## 8. Targeting Cholesterol Metabolism in AML Therapy

Standard induction therapy for AML consists of continuous Ara-C (100–200 mg/m^2^ for 7 days) combined with an anthracycline: idarubicin (IDA; 12 mg/m^2^) or DNR (45–60 mg/m^2^) for 3 days, commonly known as the “7 + 3” regimen. Patients with adverse or intermediate cytogenetic risk often require an intensified dose of Ara-C. Conversely, elderly or unfit patients, due to comorbidities and reduced tolerance to intensive chemotherapy, are generally treated with low-dose Ara-C or hypomethylating agents (HMAs), including decitabine (DAC) or 5-azacitidine (AZA), often in combination with therapies targeting FLT3, KIT, IDH1, or IDH2. Acute promyelocytic leukemia (APL) follows a different therapeutic paradigm based on fully trans retinoic acid (ATRA), combined with chemotherapy, arsenic trioxide (ATO), or ATRA plus ATO with minimal or no chemotherapy. Some cholesterol-lowering agents have shown intrinsic cytotoxicity in several AML models and may further sensitize specific AML subtypes to standard treatments (Table 2) [30].

### 8.1. Statins

Statins inhibit HMGCR, the rate-limiting enzyme of the mevalonate pathway, making them effective cholesterol-lowering agents widely used for the prevention and treatment of cardiovascular disease [102]. Increasing evidence supports their repurposing in cancer therapy, as statins induce apoptosis and ferroptosis, promote differentiation, modulate autophagy, and remodel the tumor microenvironment toward a more antitumor state (Table 2) [103].

**Table 2:** Cholesterol-lowering agents as antileukemic and chemosensitizing strategies in acute myeloid leukemia.

Drug	Main Target	Effects on Cancer Cells	Cholesterol Related Mechanisms	Clinical Phase	Cancer Therapy Complementarity	References
Simvastatin	HMGCR	Apoptosis, ferroptosis, reduced proliferation, reduced migration, leukemic stem cell targeting, chemosensitization	Inhibits mevalonate pathway, reduces geranylgeranylation of RAS and RHEB, impairs prenylation and vesicular trafficking	Preclinical Phase I	Ara-C IDA	[87] [104] [105]
Atorvastatin	HMGCR	Apoptosis, differentiation, ferroptosis, sensitization	Inhibits mevalonate pathway, activates RAC1 and CDC42 JNK signaling, reduces prenylation	Preclinical	ATRA	[106]
Fluvastatin	HMGCR	Apoptosis, differentiation, suppression of leukemic progenitors	Inhibits mevalonate pathway, reduces farnesylation, activates JNK pathway	Preclinical	ATRA CHR2863	[74] [106]
Lovastatin	HMGCR	Apoptosis, reduced ABCB1 expression, leukemic stem cell targeting, chemosensitization	Inhibits geranylgeranylation, blocks prenylation, apoptosis rescued by mevalonate	Preclinical	Conventional chemotherapy	[107] [108]
Pravastatin	HMGCR	Moderate apoptosis, reduced ABCB1, chemosensitization	Inhibits mevalonate pathway and prenylation	Phase I Phase II	Ara-C IDA	[109]
Zoledronic acid	FDPS	Growth arrest, apoptosis, enhanced γδ T-cell cytotoxicity	Blocks synthesis of isoprenoid intermediates required for protein prenylation	Preclinical	Potential immunotherapy combinations	[110]
Alendronate	FDPS	Growth arrest, apoptosis	Inhibits prenylation of RAS/RHO/RAB GTPases through mevalonate pathway blockade	Preclinical	Potential chemotherapy combinations	[111]
Pamidronate	FDPS	Growth arrest, apoptosis	Inhibits protein prenylation by blocking FDPS	Preclinical	Potential chemotherapy combinations	[112]
Zaragozic acid	SQS	Reverses chemoresistance, granulocytic differentiation	Prevents adaptive cholesterol accumulation induced by chemotherapy	Preclinical	Potential combination with chemotherapy	[31]
Hymeglusin	HMGCS1	Sensitizes AML cells to chemotherapy	Inhibits upstream mevalonate pathway, reducing cholesterol biosynthesis	Preclinical	Ara-C, DOX	[113]
Tamoxifen	ERα	Reduced proliferation, oxidative stress, ER stress, inhibition of IL-6/JAK2/STAT3 signaling	Inhibits terminal cholesterol biosynthesis causing 7-DHC accumulation (also inhibits EBP)	Preclinical	Not evaluated	[114]
HP-β-CyD	Cholesterol depletion	G2/M arrest, apoptosis, prolonged survival in mice	Directly removes intracellular cholesterol	Preclinical	Potential chemotherapy combinations	[115]
Dendrogenin A (DDA)	LXR	Sensitization to Ara-C, autophagy induction	Activates LXR signaling, induces NR4A1/NR4A3, LC3 and TFEB transcriptional programs	Preclinical	Ara-C	[116] [117]

In AML, these effects are thought to arise primarily from disruption of mevalonate-derived metabolic intermediates rather than cholesterol depletion alone, highlighting the mevalonate pathway as a metabolic vulnerability that can be therapeutically exploited [10,118].

Extensive preclinical evidence demonstrates that lipophilic statins—including simvastatin, atorvastatin, lovastatin, and fluvastatin—exert broad antileukemic activity across AML cell lines and primary patient samples. A chemical screening study identified atorvastatin and simvastatin as potent inhibitors of primary AML cells representing multiple cytogenetic subtypes, with the greatest sensitivity observed in AML with core-binding factor abnormalities [inv(16) and t(8;21)], AML M4Eo, and samples with low bone marrow blast counts [86]. Likewise, simvastatin significantly inhibited proliferation and colony formation of HL-60 and AML-2 cells while potentiating the growth-inhibitory activity of Ara-C [87]. Simvastatin also displayed heterogeneous activity among AML models, with NRAS G12D-mutated THP-1 cells showing marked sensitivity associated with profound inhibition of RAS-dependent signaling pathways [104]. Beyond its antiproliferative effects, simvastatin suppressed migration and matrix metalloproteinase-9 (MMP-9) secretion in THP-1 cells through inhibition of protein geranylgeranylation [119].

Similar antileukemic activity has been reported for lovastatin, which induces apoptosis in several AML cell lines at clinically relevant concentrations [107], and for atorvastatin and fluvastatin, which promote both apoptosis and differentiation of APL cells while enhancing ATRA-induced differentiation and partially overcoming ATRA resistance [106]. Moreover, several lipophilic statins induce monocytic differentiation in non-APL AML cells through inhibition of the mevalonate pathway and activation of differentiation-associated transcriptional programs involving Krueppel-like factor 4 (KLF4) and dihydropyrimidinase-like 2 (DPYSL2A) [120].

Mechanistic studies consistently indicate that inhibition of protein prenylation represents the principal antileukemic mechanism of statins in AML. Genetic and pharmacological analyses demonstrated that statin sensitivity depends on the disruption of protein geranylgeranylation, particularly affecting small GTPases involved in intracellular signaling and vesicular trafficking, including members of the RAS, RAB, and RHEB families [74,86]. Consistent with this mechanism, statins alter vesicular transport in primary AML samples, and statin-sensitive leukemias exhibit transcriptional signatures indicative of enhanced vesicular trafficking activity [86]. Likewise, the synergistic cytotoxicity observed between aminopeptidase inhibitors (CHR2863 and bestatin) and multiple statins—including simvastatin, fluvastatin, lovastatin, and pravastatin—is completely reversed by supplementation with mevalonate or FPP, confirming that disruption of the mevalonate pathway underlies this therapeutic interaction [74]. Similarly, lovastatin-induced apoptosis is prevented by supplementation with mevalonate and downstream mevalonate pathway metabolites, whereas inhibition of protein geranylgeranylation, rather than depletion of cholesterol or other sterol intermediates, is required for efficient induction of leukemic cell death [108,121].

Beyond directly suppressing leukemic proliferation, statins also enhance the activity of established AML therapies. Simvastatin potentiates the cytotoxic effects of Ara-C in AML cell lines [87], whereas fluvastatin enhances the antileukemic activity of ATRA and suppresses colony formation by primary AML progenitors [106]. Lovastatin further sensitizes CD34+ LSC-enriched populations to conventional chemotherapy, while statin treatment reduces the expression of the multidrug resistance transporter ABCB1 in KG1a cells, suggesting an additional mechanism for overcoming drug resistance [122].

The antileukemic activity of statins has also been supported by in vivo studies and early-phase clinical trials. Continuous administration of simvastatin significantly reduced leukemic burden in the bone marrow and spleen of mice transplanted with HL-60 cells while largely preserving normal hematopoietic progenitors [123,124]. In patients with AML, short-term administration of high-dose simvastatin enhanced chemotherapy sensitivity in a subset of patients, an effect associated with inhibition of protein geranylgeranylation [105]. Similarly, the addition of pravastatin to IDA plus high-dose Ara-C proved safe in a phase I clinical trial and achieved complete remission or complete remission with incomplete platelet recovery in approximately 73% of patients, particularly at higher pravastatin doses [125]. A subsequent phase II study in poor-risk AML demonstrated a 30% overall response rate and enabled approximately one-quarter of patients to proceed to allogeneic hematopoietic stem cell transplantation, with transplanted patients achieving a median overall survival of 27.1 months [109].

Collectively, these findings identify statins as promising metabolic adjuvants in AML by targeting the mevalonate pathway to impair protein prenylation, suppress leukemic growth, promote differentiation, and enhance therapeutic responses. Although clinical evidence remains limited and many preclinical studies have used suprapharmacological statin concentrations, the convergence of mechanistic and translational data provides a strong rationale for evaluating statins in combination with current AML therapies.

### 8.2. Other Cholesterol-Targeting Agents

Beyond statins, several additional pharmacological strategies targeting different steps of cholesterol metabolism have shown promising preclinical activity in AML (Table 2). Although these approaches remain less extensively investigated, they further support cholesterol homeostasis as a therapeutically exploitable vulnerability.

Targeting the mevalonate pathway downstream of HMGCR represents one such approach. Nitrogen-containing bisphosphonates, including zoledronic acid, alendronate, and pamidronate, inhibit FDPS, preventing the synthesis of the isoprenoid intermediates required for protein prenylation. Consequently, prenylation-dependent signaling mediated by members of the RAS, RHO, RAC, and RAB GTPase families is disrupted, leading to growth arrest and apoptosis in AML cells [126]. Furthermore, according to a preprint, the inhibition of geranylgeranyl diphosphate synthase (GGDPS) with CML-07-119 exhibits potent antileukemic activity, particularly in TP53-mutated AML, while nitrogen-containing bisphosphonates also enhance γδ T-cell-mediated cytotoxicity against primary AML blasts [127,128]. Pharmacological inhibition of HMGCS1 with hymeglusin likewise sensitizes AML cells to Ara-C and DOX, further supporting the therapeutic value of targeting the mevalonate pathway upstream of cholesterol synthesis [55].

Some studies have also investigated inhibition of enzymes committed specifically to cholesterol biosynthesis. Computational metabolic analyses identified SQS as a candidate target for reversing chemoresistance in AML, whereas pharmacological inhibition with zaragozic acid prevented the adaptive cholesterol accumulation induced by chemotherapy and promoted granulocytic differentiation of HL-60 cells [31,89,129]. More recently, inhibition of the terminal cholesterol biosynthetic enzyme 7-dehydrocholesterol (7-DHC) reductase (DHCR7), either by genetic silencing or pharmacological treatment with tamoxifen, reduced AML cell proliferation, promoted oxidative and endoplasmic reticulum stress through accumulation of 7-DHC, and suppressed IL-6/JAK2/STAT3 signaling in preclinical models [114]. However, because tamoxifen also inhibits emopamil-binding protein (EBP) and affects lysosomal function, its precise mechanism of action in AML remains controversial.

Additional strategies directly targeting cholesterol availability have also shown encouraging preclinical activity. Cholesterol depletion with 2-hydroxypropyl-β-cyclodextrin (HP-β-CyD) markedly reduced intracellular cholesterol, induced G2/M cell-cycle arrest and apoptosis, and significantly prolonged survival in murine AML models [115]. Likewise, disruption of scavenger receptor class B type 1 (SR-B1)-mediated cholesterol transport using synthetic high-density lipoprotein (HDL) nanoparticles impaired cholesterol homeostasis, depleted glutathione peroxidase 4 (GPX4), and induced predominantly ferroptotic cell death, while also promoting granulocytic differentiation. Notably, HDL nanoparticles enhanced the activity of Ara-C, venetoclax, and gilteritinib, particularly in FLT3-mutated AML models [54].

Finally, cholesterol esterification has emerged as another potential therapeutic target. In solid cancers, pharmacological SOAT1 inhibition increases free cholesterol accumulation, promotes apoptosis, and enhances CD8^+^ T-cell function by increasing plasma membrane cholesterol availability [130]. Although direct evidence in AML remains limited, these findings suggest that targeting cholesterol storage may complement strategies directed against cholesterol biosynthesis. In addition, AZA has recently been shown to perturb cholesterol homeostasis indirectly by reducing the expression of key lipid metabolic regulators, including PCSK9, HMGCR, and FASN, while simultaneously altering intracellular cholesterol distribution and redirecting lipid metabolism toward triacylglycerol synthesis and lipid droplet accumulation [131]. Although these effects are unlikely to represent the primary mechanism of action of AZA, they suggest that modulation of cholesterol metabolism may contribute to its antileukemic activity.

Beyond inhibiting cholesterol biosynthesis or transport, cholesterol-derived metabolites themselves may also possess therapeutic potential. Dendrogenin A (DDA), an endogenous cholesterol metabolite that is reduced in several malignancies, binds and activates LXRs, inducing a transcriptional program involving nuclear receptor subfamily 4 immunity group A member 1 (NR4A1) and member 3 (NR4A3), microtubule-associated protein 1A/1B-light chain 3 (LC3), and transcription factor EB (TFEB) [132]. In AML, DDA sensitized both leukemic cell lines and primary patient samples to Ara-C, supporting the concept that pharmacological exploitation of endogenous cholesterol metabolites may represent an alternative strategy to target cholesterol-dependent metabolic vulnerabilities [116,117].

### 8.3. Possible Side Effects of Cholesterol-Targeting Agents in AML Patients

Although cholesterol-targeting strategies enhance chemosensitivity and overcome metabolic adaptation in AML, their clinical implementation requires careful consideration of treatment-related toxicities. Importantly, the safety profile varies substantially according to the targeted step of the cholesterol biosynthetic pathway and the pharmacological properties of each agent.

Statins remain the most clinically advanced approach. High-dose pravastatin combined with IDA and Ara-C was generally feasible, but treatment was associated with frequent febrile neutropenia, severe diarrhea and occasional early treatment-related mortality secondary to infectious complications and multiorgan failure, particularly during intensive induction therapy [125,133]. Lipophilic statins, including simvastatin, lovastatin and fluvastatin, carry additional risks of dose-dependent myopathy, rhabdomyolysis, hepatotoxicity and renal injury, reflecting the systemic inhibition of the mevalonate pathway rather than leukemia-specific toxicity [103]. While atorvastatin appears less toxic toward normal hematopoietic cells in preclinical models, its clinical safety in AML remains insufficiently established [134].

Nitrogen-containing bisphosphonates introduce a distinct toxicity spectrum characterized by renal dysfunction, acute-phase reactions, hypocalcemia and, with prolonged administration, osteonecrosis, particularly of the jaw [135,136]. These adverse effects may be amplified in AML patients receiving nephrotoxic chemotherapy or experiencing tumor lysis syndrome and sepsis [137].

More selective metabolic inhibitors may offer an improved therapeutic window. Hymeglusin enhances the activity of venetoclax and conventional chemotherapy without increasing myelosuppression or toxicity in normal hematopoietic cells in preclinical studies [138]. Similarly, DDA and HP-β-CyD exhibit preferential antileukemic activity while largely sparing normal bone marrow cells, although further studies are required to define their long-term safety in humans [119,132].

Overall, current evidence suggests that selective targeting of cholesterol metabolism downstream of HMGCR, or tumor-directed delivery systems, may reduce systemic toxicity while preserving the therapeutic benefit of metabolic intervention. Nevertheless, prospective clinical studies remain essential to determine whether these promising preclinical safety profiles translate into improved tolerability in patients with AML.

## 9. Conclusions and Future Perspectives

AML exhibits a coordinated reprogramming of cholesterol metabolism that extends far beyond increased lipid availability. Throughout this review, the available evidence supports the concept that cholesterol functions as an organizational hub integrating membrane dynamics, mitochondrial homeostasis, oncogenic signaling, metabolic adaptation, and immune regulation. Rather than representing isolated metabolic alterations, enhanced cholesterol biosynthesis, receptor-mediated uptake, intracellular trafficking, and esterification form an interconnected network that enables leukemic cells to sustain proliferation, survive metabolic stress, and evade therapy (Figure 1).

This integrated network also provides a mechanistic explanation for the paradoxical coexistence of systemic hypocholesterolemia and increased intracellular cholesterol demand in AML, reinforcing cholesterol metabolism as a central regulator of leukemic biology. Emerging evidence further indicates that cholesterol reprogramming extends beyond tumor-intrinsic metabolism to influence immune crosstalk within the bone marrow microenvironment by regulating immune checkpoint expression, stromal remodeling, nutrient availability, and immune-cell metabolic fitness. Together, these observations identify cholesterol metabolism as a biological interface linking leukemic cell metabolism with immune dysfunction.

Despite these advances, several important knowledge gaps remain. First, although cholesterol accumulation in LSCs has been associated with therapy resistance, the mechanisms governing cholesterol trafficking among mitochondria, lipid droplets, and plasma membranes in LSCs remain poorly understood. Second, the extent to which recurrent AML-associated genetic alterations—including FLT3-ITD, NPM1, IDH1/2, TET2, RUNX1, CEBPA, TP53, and KMT2A rearrangements—generate distinct cholesterol metabolic dependencies has not been systematically investigated. Third, although increasing evidence suggests that cholesterol regulates immune dysfunction, most mechanistic evidence has been extrapolated from solid tumors, whereas direct experimental validation in AML remains limited. Finally, reliable biomarkers capable of identifying cholesterol-dependent AML subtypes or predicting response to cholesterol-targeted therapies are still lacking.

Among these unresolved questions, three priorities appear particularly important. The first is defining cholesterol metabolism in LSCs, as these cells largely determine relapse and treatment failure. The second is establishing genotype-specific cholesterol vulnerabilities that could enable precision metabolic therapies. The third is clarifying how cholesterol metabolism modulates immune-cell function within the bone marrow niche, particularly during treatment with hypomethylating agents, venetoclax, immune checkpoint inhibitors, and cellular immunotherapies.

Addressing these questions will require more sophisticated experimental approaches than those currently available. Single-cell and spatial lipidomics combined with isotope-tracing strategies could define cholesterol flux at cellular and subcellular resolution throughout disease progression and treatment. Organoid systems and patient-derived xenografts incorporating both leukemic and immune compartments would enable mechanistic investigation of cholesterol-dependent immune regulation under physiologically relevant conditions. Likewise, CRISPR-based functional genomic screens focused on cholesterol biosynthesis, transport, esterification, and intracellular trafficking across genetically defined AML models could identify subtype-specific metabolic liabilities. Finally, prospective clinical studies integrating transcriptomics, lipidomics, metabolomics, and circulating lipid biomarkers with therapeutic response will be essential for developing predictive biomarkers and rationally selecting patients who may benefit from cholesterol-targeted interventions.

From a therapeutic perspective, future studies should move beyond evaluating cholesterol-lowering drugs as isolated agents and instead define rational combination strategies. In particular, combining inhibitors of cholesterol metabolism with venetoclax-based regimens, FLT3 inhibitors, IDH inhibitors, hypomethylating agents, or emerging immunotherapies may simultaneously impair leukemic metabolism, reduce stem-cell persistence, and restore antitumor immunity. The temporal dynamics of cholesterol metabolism also deserve further investigation, including whether circadian regulation of the mevalonate pathway could be exploited to optimize treatment scheduling.

Overall, the evidence reviewed here supports a revised conceptual framework in which cholesterol reprogramming constitutes a central organizing principle of AML pathogenesis rather than a secondary metabolic adaptation. By integrating tumor-intrinsic metabolism with immune crosstalk, cholesterol metabolism emerges as a promising source of biologically meaningful biomarkers and therapeutically actionable vulnerabilities. A more comprehensive understanding of these interconnected mechanisms is likely to facilitate the development of metabolism-based precision therapies capable of improving clinical outcomes in this highly heterogeneous disease.

Overall, the evidence reviewed here supports cholesterol metabolism as a central regulator of AML biology and a promising source of biologically meaningful biomarkers and therapeutically actionable vulnerabilities. Rather than representing an isolated metabolic alteration, cholesterol reprogramming integrates leukemic metabolism with immune regulation, providing a rationale for metabolism-based precision therapies. Future studies combining genomic, lipidomic, metabolomic, and immune profiling will be essential to identify patients most likely to benefit from cholesterol-targeted interventions and to translate these findings into improved clinical outcomes.

## Data Availability

No new data were created or analyzed in this study. Data sharing is not applicable to this article.

## Abbreviations

The following abbreviations are used in this manuscript:

25-OHC	25-Hydroxycholesterol
27-OHC	27-Hydroxycholesterol
7β-OHC	7β-Hydroxycholesterol
ABCB1	ATP-binding cassette subfamily B member 1
ABCA1	ATP-binding cassette subfamily A member 1
ABCG1	ATP-binding cassette subfamily G member 1
ABCG5	ATP-binding cassette subfamily G member 5
ABCG8	ATP-binding cassette subfamily G member 8
AKT	Protein kinase B
AML	Acute myeloid leukemia
AMPK	AMP-activated protein kinase
AP-1	Activator protein-1
APL	Acute promyelocytic leukemia
Ara-C	Cytarabine
ARG1	Arginase-1
ATO	Arsenic trioxide
ATRA	All-trans retinoic acid
AZA	Azacitidine
CBS	Cystathionine β-synthase
CD36	Cluster of differentiation 36
CES1	Carboxylesterase 1
CN-AML	Cytogenetically normal acute myeloid leukemia
CTLA-4	Cytotoxic T-lymphocyte-associated protein 4
CYP	Cytochrome P450
CYP7B1	Cytochrome P450 family 7 subfamily B member 1
DAC	Decitabine
DHCR7	7-Dehydrocholesterol reductase
DMSO	Dimethyl sulfoxide
DNR	Daunorubicin
DOX	Doxorubicin
DPYSL2A	Dihydropyrimidinase-like 2A
EBP	Emopamil-binding protein
EGF	Epidermal growth factor
EGFR	Epidermal growth factor receptor
EOMES	Eomesodermin
FAB	French–American–British classification
FAO	Fatty acid oxidation
FAT	Fatty acid translocase
FDPS	Farnesyl diphosphate synthase
FLT-1	Fms-like tyrosine kinase 1 (VEGFR1)
FLT3	Fms-like tyrosine kinase 3
FLT3-ITD	Fms-like tyrosine kinase 3 internal tandem duplication
FPP	Farnesyl pyrophosphate
GGPS	Geranylgeranyl diphosphate synthase
GGPP	Geranylgeranyl pyrophosphate
GPCR	G protein-coupled receptor
GPX4	Glutathione peroxidase 4
GSH	Reduced glutathione
HDL	High-density lipoprotein
HMG-CoA	3-Hydroxy-3-methylglutaryl-coenzyme A
HMGCR	3-Hydroxy-3-methylglutaryl-CoA reductase
HMGCS1	3-Hydroxy-3-methylglutaryl-CoA synthase 1
HMGCS2	3-Hydroxy-3-methylglutaryl-CoA synthase 2
H_2_S	Hydrogen sulfide
HP-β-CyD	2-Hydroxypropyl-β-cyclodextrin
IDA	Idarubicin
IDH	Isocitrate dehydrogenase
IDI1	Isopentenyl-diphosphate delta isomerase 1
IFN-γ	Interferon gamma
IL	Interleukin
INSIG	Insulin-induced gene
IPP	Isopentenyl pyrophosphate
JAK	Janus kinase
LDL	Low-density lipoprotein
LDLR	Low-density lipoprotein receptor
LIMA1	LIM domain and actin-binding protein 1
LSC	Leukemic stem cell
LSS	Lanosterol synthase
LXR	Liver X receptor
LYCHOS	Lysosomal cholesterol sensing protein
MMP-9	Matrix metalloproteinase-9
MSC	Mesenchymal stromal cell
mTOR	Mechanistic target of rapamycin
mTORC1	Mechanistic target of rapamycin complex 1
MVK	Mevalonate kinase
MVD	Mevalonate diphosphate decarboxylase
MYLIP	Myosin regulatory light chain interacting protein
NADP^+^	Nicotinamide adenine dinucleotide phosphate (oxidized)
NADPH	Nicotinamide adenine dinucleotide phosphate (reduced)
NF-κB	Nuclear factor κB
NK	Natural killer
NPC1L1	Niemann-Pick C1-like 1
NRF1	Nuclear factor erythroid 2-related factor 1
OXPHOS	Oxidative phosphorylation
PCSK9	Proprotein convertase subtilisin/kexin type 9
PD-1	Programmed cell death protein 1
PD-L1	Programmed death-ligand 1
PI3K	Phosphoinositide 3-kinase
PlGF	Placenta growth factor
PMA	Phorbol 12-myristate 13-acetate
PMVK	Phosphomevalonate kinase
PPAR	Peroxisome proliferator-activated receptor
PRDX	Peroxiredoxin
ROS	Reactive oxygen species
SCAP	SREBP cleavage-activating protein
SOAT1	Sterol O-acyltransferase 1
SOD	Superoxide dismutase
SQLE	Squalene epoxidase
SQS	Squalene synthase
SR-B1	Scavenger receptor class B type 1
SREBP	Sterol regulatory element-binding protein
SREBP2	Sterol regulatory element-binding protein 2
STAT	Signal transducer and activator of transcription
TBX21	T-box transcription factor 21
THBS1	Thrombospondin-1
TIM-3	T-cell immunoglobulin and mucin-domain-containing protein 3
TNF-α	Tumor necrosis factor alpha
TPA	12-O-Tetradecanoylphorbol-13-acetate
VEGF	Vascular endothelial growth factor
VLDL	Very-low-density lipoprotein
